# Can Pharmacogenomic Testing Aid Psychiatric Medication Prescriptions?

**DOI:** 10.1192/bjo.2026.11938

**Published:** 2026-06-30

**Authors:** Lauren Varney, Marius Cotic, Agostina Secchi, Myles Howard, Elvira Bramon

**Affiliations:** 1 University College London, London, United Kingdom; 2 Kent and Medway Mental Health NHS Trust, Kent, United Kingdom; 3 North London NHS Foundation Trust, London, United Kingdom

## Abstract

**Aims:**

Various antipsychotics and antidepressants have pharmacogenomic guidelines available from international sources, recommending dose adjustments and/or alternative drug selection based on a patient’s genetic profile. However, pharmacogenomic implementation remains limited in UK healthcare.

**Methods:**

Genetics and Environment in Mental Health Study (GEMS) is a prospective study of pharmacogenomic testing in psychosis; the first in the UK. Adults taking an antipsychotic medication have been recruited across England. Participants provided a DNA sample for genotyping on a multi-gene panel, selected from evidence-based pharmacogenomic guidelines. Pharmacogenomic reports, displaying the participants’ genetic profile and any alterations to usual prescribing guidelines were delivered to the prescribing clinician. Any changes to the participants’ prescription were at the discretion of the clinician.

**Results:**

A diverse sample of 584 people taking an antipsychotic medication has been recruited so far, consisting of participants aged 18-82 years, with an even split of male and female participants, and 32.5% of the sample from Black, Asian, and Minority Ethnic (BAME) backgrounds.

Across the four genes with pharmacogenomic guidelines in psychiatry (*CYP2D6*, *CYP2C19*, *CYP2B6*, and *CYP3A4*), 90.4% of the sample carried at least one actionable variant. At the time of recruitment, 20.9% of participants carried a pharmacogenomic variant that (based on current guidelines) was actionable for the antipsychotic and/or antidepressant they were currently prescribed. By medication class, 6.8% were taking an antipsychotic and 15.1% were taking an antidepressant for which they had a pharmacogenomic recommendation. A further 69.5% of the sample would have a recommendation for at least one antipsychotic or antidepressant medication.

Based on the estimated population prevalence of pharmacogenomic actionable variants and prescription data in England, approximately 45% of people would be expected to carry an actionable variant for antipsychotic medications and at least 61% would be expected to carry an actionable variant for antidepressants. This equates to approximately 285,000 people currently taking an antipsychotic medication that could have a pharmacogenomic recommendation and at least 4.2 million people taking an antidepressant with a pharmacogenomic recommendation in England.

**Conclusion:**

Pharmacogenomics has the potential to personalise medication prescriptions and improve patient outcomes. Workforce training and implementation barriers present a challenge. However, in line with the NHS 10 Year Plan goal of 50% of healthcare interventions being genomics-informed by 2035, psychiatry is one of the most promising areas for the implementation of pharmacogenomic-guided prescribing.

